# Sex Disparity in Stroke Mortality among Adults: A Time Series Analysis in the Greater Vitoria Region, Brazil (2000–2021)

**DOI:** 10.3390/epidemiologia5030029

**Published:** 2024-07-17

**Authors:** Orivaldo Florencio de Souza, Alexandre Castelo Branco Araújo, Lorenna Baião Vieira, José Alexandre Bachur, Alberto Grover Prado Lopez, Thiago Gomes Gonçalves, Luiz Carlos de Abreu

**Affiliations:** 1 Postgraduate Program in Nutrition and Health, Federal University of Espírito Santo, Vitoria 29043-900, Brazil; orivaldo.souza@ufac.br; 2 Postgraduate Program in Health Sciences, Federal University of Acre, Rio Branco 69915-900, Brazil; 3 Health Sciences Center, Federal University of Espírito Santo, Vitoria 29043-900, Brazil; 4 Postgraduate Program in Public Health, Federal University of Espírito Santo Vitoria 29043-900, Brazil; 5 Medical School, University of Franca, Franca 29102-920, Brazil; 6 Medical Sector, Federal University of Acre, Rio Branco 69915-900, Brazil; 7 Postgraduate Program in Medical Sciences, University of Sao Paulo, São Paulo 01246-903, Brazil

**Keywords:** stroke, cerebrovascular disorders, epidemiologic measurements, time series

## Abstract

The disparity between the sexes in stroke mortality has been demonstrated in people from different locations. The objective of this study was to analyze the disparity between sexes in stroke mortality in adults in the metropolitan area of Greater Vitoria between 2000 and 2021. Ecological time series design was conducted with a database of the Brazilian Health System Informatics Department. The annual percentage change and average annual percentage change were calculated through joinpoint regression. Pairwise comparisons using parallelism and coincidence tests were applied to compare temporal trends between men and women. Men had higher mortality rates in most years between 2000 and 2021. In contrast, women had higher proportional mortality values in all years evaluated from 2000 to 2021. The paired comparison revealed a disparity between the sexes in the proportional mortality time series (parallelism test: *p* = 0.003; coincidence test: *p* < 0.001). However, the time series of the mortality rates showed no disparity between the sexes (parallelism test: *p* = 0.114; coincidence test: *p* = 0.093). From 2000 to 2021, there was a disparity in proportional mortality from stroke between the sexes of the population in the metropolitan area of Greater Vitoria, Brazil. However, the time series of mortality rates between the sexes did not reveal any disparity in the study period.

## 1. Introduction

In 2022, there were 6,552,724 stroke deaths worldwide, of which 51% were men [1]. Globally, data from the Global Burden of Disease have revealed that stroke is one of the leading causes of death in men and women [2,3]. Stroke has shown a decreasing trend in recent decades, especially during the COVID-19 pandemic, with a reduction in ranking from the second to the third leading cause of death worldwide [3]. Furthermore, there is a higher incidence of stroke in women, but the mortality rate is higher in men [1]. In Latin America and the Caribbean, stroke was the third leading cause of death in 2021, with 279,000 deaths [3]. In recent decades, the temporal trend in the stroke mortality rate in Latin America has seen a higher decline in women (−2.4%) than in men (−1.4%) [4]. The health ministries of 13 Latin American countries have declared priorities to reduce stroke-related deaths [5]. Therefore, studies on the temporal trends in stroke in different locations in Latin America are necessary to obtain a satisfactory contextual understanding.

Disparities in stroke mortality between sexes have been discussed and published in the scientific literature [6,7]. The decrease in the mortality rate and proportional mortality has different magnitudes between men and women in various countries [8,9,10,11]. In the southeast region of Brazil, the cumulative death count was higher in men between 1997 and 2012, but proportional mortality was higher in women [12]. Studies report a higher prevalence of non-focal or atypical symptoms at the onset of stroke in women compared to men [13,14]. The consequence of this mistaken diagnosis can lead to delayed treatment and increased stroke mortality in women [15].

Socioeconomic and health inequalities between individuals from different locations can result in different magnitudes of the annual percentage change in stroke mortality between sexes [16,17]. Updated information on the temporal trend of stroke mortality for both sexes has been published for the north and northeast regions of Brazil [6,7]. The disease burden, as evidenced by the disability-adjusted life years indicator, was reported for several populations in Brazil, including the southeast region [18]. However, to the best of our knowledge, there are no updated studies on sex disparities in stroke mortality among adults in the metropolitan area of Greater Vitoria, located in the southeast region of Brazil. Therefore, our objective was to analyze the disparity between sexes in stroke mortality in adults in the metropolitan area of Greater Vitoria between 2000 and 2021.

## 2. Materials and Methods

### 2.1. Study Design, Location, and Population

This is an ecological time series design from 2000 to 2021. This study was based on secondary data from the adult population of the metropolitan area of Greater Vitoria, Brazil. In 2021, the metropolitan area of Greater Vitoria had a Human Development Index of 0.796, a population of 2,033,067 inhabitants, and a density of 872.18 inhabitants per km2 [19].

### 2.2. Data Source

The data source was the public access database of the Department of Informatics of the Unified Health System (DATASUS), an organization linked to the Brazilian Ministry of Health. The number of stroke deaths and the number of deaths from all causes was accessed through the ‘Vital Statistics’ tab on the website https://datasus.saude.gov.br/informacoes-de-saude-tabnet/ (accessed on 10 November 2023). Information on the resident population by sex and year was accessed through the ‘Demography and Socioeconomics’ tab on the website https://datasus.saude.gov.br/populacao-residente (accessed on 10 November 2023). Trained researchers performed data extraction. Discrepancies in data extraction between researchers were resolved by consensus through repeated extractions.

### 2.3. Eligibility Criteria

The inclusion criteria were adult death from stroke and the deceased’s reported place of residence being in the metropolitan area of Greater Vitoria. The exclusion criterion was registered notification for the ignored sex variable.

### 2.4. Study Variable

The study variable was stroke as the underlying cause of death. According to the International Classification of Diseases version 10, stroke was classified as subarachnoid hemorrhage (I60), intracerebral hemorrhage (I61), cerebral infarction (I63), and stroke not classified as ischemic or hemorrhagic (I64). The stroke variable was stratified by sex and year.

### 2.5. Data Analysis

For each sex and year between 2000 and 2021, the mortality rate and proportional mortality due to stroke and the percentage difference between the sexes were calculated using a Microsoft Excel spreadsheet. The mortality rate was calculated by dividing the number of stroke deaths by the population; the division quotient was multiplied by 100,000 inhabitants. The proportional mortality was calculated by dividing the number of stroke deaths by the number of deaths from all causes; the division quotient was multiplied by 100. To calculate the percentage difference in the mortality rate and the proportional mortality from stroke between men and women, the values for women were subtracted from those for men. Next, the difference obtained was divided by the values for men. Subsequently, the quotient was multiplied by 100.

Joinpoint regression models were applied to identify change points in the time series and the trend in each segment from 2000 to 2021, with the help of the Joinpoint Regression Program (version 5.0.2, 2023) [20]. The weighted Bayesian information criterion method was applied to select the models. In the joinpoint regression models with a *p*-value < 0.05, the hypothesis of an annual percentage change or an average annual percentage change was accepted. The disparity between the sexes in the temporal trend in the mortality rate and proportional mortality was assessed by pairwise comparison using parallelism and coincidence tests. *p*-values < 0.05 rejected the hypothesis of parallelism or coincidence. Furthermore, the difference in the average annual percentage change between the sexes was calculated. Therefore, a *p*-value < 0.05 accepted the hypothesis of a statistically significant difference in the average annual percentage change between the sexes.

## 3. Results

From 2000 to 2021, there were 12,008 stroke deaths in adults aged 20 years or older in the metropolitan area of Greater Vitoria. The exclusions were two cases that were noted as ignored in the sex variable. In the time series, there were 6154 (51.3%) deaths from stroke in women.

In most years between 2000 and 2021, the stroke mortality rate was higher in men than in women. Men had the highest mortality rate in 2000 and the lowest in 2016. Women had the highest mortality rate in 2002 and the lowest in 2017. Throughout the study period, proportional mortality was higher in women. Furthermore, women showed high proportional mortality between 2000 and 2003. There was a decrease in proportional mortality values between 2019 and 2021 for both sexes (Table 1).

**Figure 1:**
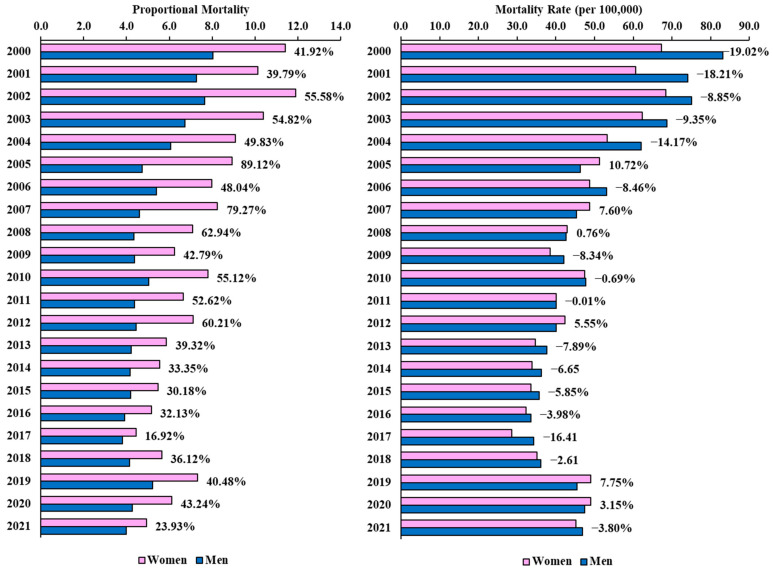
Percentage difference between the sexes in the mortality rate (per 100,000 inhabitants) and proportional mortality due to stroke from 2000 to 2021.

Figure 1 shows the percentage difference between women and men in the mortality rate and proportional mortality from stroke. In 2005, there was a high percentage difference in the mortality rate (10.72%) in favor of women. From 2006 onward, the percentage difference in the mortality rate between the sexes was less than 10%, except in 2017. Between 2000 and 2012, proportional mortality from stroke was 40% higher in women than in men. In 2005, there was the largest proportional difference in proportional mortality (89.12%) in favor of women.

Table 2 shows the annual percentage change, the average annual percentage change, and the difference between the average annual percentage changes in the adult mortality rates due to stroke. Men had a reduction in the average annual percentage change in the mortality rate from stroke of −2.4 (*p* < 0.001), whereas women showed a stationary trend during the study period. In the 2000–2021 period, the difference in the average annual percentage change in the mortality rate between the sexes of 1.3 did not reveal statistical significance (*p* = 0.275). The annual percentage change revealed a statistically significant decrease (*p* < 0.05) in the mortality rate for both sexes from 2000 to 2017. Men and women showed an increase in mortality rates from 2017 to 2021, with an annual percentage change of 10.3% (*p* = 0.011) and 14.4% (*p* = 0.003), respectively. The parallelism test showed that the mortality rate changed in parallel (*p* = 0.114) between the sexes between 2000 and 2021. The coincidence test revealed a similarity (*p* = 0.093) in the temporal trend between the sexes.

Table 3 presents the annual percentage change, the average annual percentage change, and the difference between the average annual percentage changes in proportional mortality from stroke in adults. Men and women had decreases in the average annual percentage change in proportional mortality of −3.3 (*p* = 0.002) and −3.1 (*p* < 0.001), respectively. From 2000 to 2021, there was a difference in the average annual percentage change in proportional mortality between men and women of 0.2. Therefore, this 0.2 difference in the average annual percentage change between the sexes in proportional mortality did not show statistical significance (*p* = 0.869). The joinpoint regression model revealed two segments in the annual percentage change in proportional mortality for both sexes, but at different periods. For men, the decreasing trend in proportional mortality (annual percentage change: −8.3%; *p* < 0.001) occurred between 2000 and 2016, followed by stability until 2021. In women, there was a decrease in proportional mortality (annual percentage change: −4.9%; *p* < 0.001) from 2000 to 2007, followed by stability until 2021. The parallelism test rejected the hypothesis (*p* = 0.003) of a parallel change in proportional mortality between men and women during the study period. Furthermore, the coincidence test revealed a disparity (*p* < 0.001) in the temporal trend between the sexes.

## 4. Discussion

A disparity in the temporal trend in proportional mortality occurred between the sexes from 2000 to 2021. The approximate values for the average annual percentage change in mortality rate and proportional mortality between men and women revealed that the variations were similar between 2000 and 2021. However, proportional mortality revealed different trends between the sexes in the temporal series. Thus, there was no parallelism in the change points and slopes of the lines between the joinpoint regression models for men and women in proportional mortality. Furthermore, there was no coincidence in the proportional mortality values between the sexes. Mortality rates between men and women showed a parallel and coincident temporal trend.

Throughout the time series, the mortality rates for men and women changed in parallel. Furthermore, there was a sharp decline for both sexes between 2000 and 2017. Similarly, global stroke data also showed a decrease in the mortality rate [2]. In several Brazilian states, there was also a decrease in stroke mortality rates for both sexes [6,12]. This decrease in stroke mortality rates may be due to government policies designed to control factors associated with stroke [21,22,23]. Furthermore, in on-site observation, the implementation of a specialized health unit for the treatment of stroke patients in the metropolitan area of Greater Victoria may have improved the diagnosis of stroke and prevented death. Consequently, it may have contributed to the reduction in the mortality rate due to stroke for both sexes in the metropolitan area of Greater Vitoria.

During the COVID-19 pandemic, there was a decrease in hospital admissions for stroke [24,25], with a high incidence of severe cases [26,27]. In most Latin American countries, stroke hospital admissions were lower during the COVID-19 pandemic than in 2019 [28]. However, there was an increase in the number of hospital admissions 48 h after the onset of stroke [28]. Similarly to the rest of the world, the incidence of COVID-19 was high in the population of the southeast region of Brazil [29,30,31]. For both sexes, in the metropolitan region of Greater Vitoria located in the southeast region of Brazil, there was an increase in the mortality rate and proportional mortality from stroke after 2016 and a subsequent decline in 2021. However, the percentage difference in the mortality rate between the sexes was less than 10%. For proportional mortality, women maintained higher values than men. Therefore, the COVID-19 pandemic did not change the profile of sex disparities in stroke mortality.

Regarding proportional mortality, there was a sex difference in the metropolitan area of Greater Vitoria, with women having a higher proportion of stroke deaths. Furthermore, women experienced a short period of decline in proportional mortality from 2000 to 2007, with a smaller magnitude of annual percentage change than men. These sex disparities can be partially attributed to the etiology of stroke, with women having a higher incidence of atrial fibrillation and hypertension than men [32]. Among the exclusive risk factors for stroke in women, the use of oral contraceptives and hormone replacement therapy in postmenopausal women is associated with stroke [33,34]. Additionally, aspects related to pregnancy and peripartum, such as pregnancy-induced hypertension, gestational diabetes, and pre-eclampsia, increase the risk of stroke in women. Previous case-fatality studies have revealed that women are more likely to die within one month after a stroke event [35]. Another study conducted in a hospital in Australia showed that one year after a stroke event, women were more likely to die than men [36]. Consequently, the probability of death increases substantially in women affected by stroke.

In Brazil, women have had a higher life expectancy than men since 2000 [37]. Elderly women are predominant in the metropolitan area of Greater Vitoria [38]. Considering that more women reach advanced age, they are therefore more susceptible to stroke [15]. This may have increased the proportion of stroke deaths in women compared with the number of deaths from all causes in the metropolitan area of Greater Vitoria.

The main strength of the study was the collection of information on stroke deaths from an information system with similar standardization during the study period. All Brazilian locations record death data in the Mortality Information System with uniformity. Subsequently, this information is transferred to DATASUS and made available as open-access data. The limitations of this study must be highlighted. Some hospitals and healthcare facilities may experience staff reductions, leading to an excessive workload. This context can expose employees to false alerts. However, in Brazil, there is permanent education for health professionals that aims at professional training and occupational health through the National Policy for Permanent Education in Health [39]. Therefore, registration failures can be mitigated by improving the quality of life of staff with better job qualifications. A small amount of information on sex was ignored, indicating the satisfactory quality of the records. The result revealed two cases omitted from the sex variable records: one in 2006 and another in 2019. However, the results did not mention whether there was a lack of registration or difficulty in determining sex for clinical reasons, such as anomalies in sexual differentiation. Therefore, we decided to apply a complete case analysis. All missing data-handling methods, including complete case analysis, perform satisfactorily when there are 1000 cases or more and missing data below 20% [40]. Considering that the present study has 12,008 deaths and a percentage of missing cases of 0.01%, we infer that the impact of applying complete case analysis on the final result is minimal.

Evidence from the present study is essential for an adequate understanding of the disparity between the sexes in the temporal trend in stroke mortality among adults in the metropolitan area of Greater Vitoria. In the evaluation of health services, stroke deaths are referred to as preventable mortality through adequate health promotion, prevention, control, and care for non-communicable diseases [41]. Therefore, stroke mortality is an acceptable epidemiological indicator of healthcare effectiveness in the metropolitan area of Greater Vitoria. Furthermore, the results of the present study highlight the need for equity in stroke healthcare in the metropolitan area of Greater Vitoria, considering the sex disparity in the magnitude of stroke mortality indicators, differences in etiologies between sexes, and stroke risk factors unique to women.

Future studies are needed to improve our understanding of the sex disparity in stroke mortality. We propose an analysis of the epidemiological case-fatality measures to describe the magnitude of deaths for the total number of stroke events by sex. In addition, survival analysis can reveal the expected duration of time until stroke-related death by sex. These suggested analyses can be stratified by age group and by subarachnoid hemorrhage, intracerebral hemorrhage, cerebral infarction, and stroke not classified as ischemic or hemorrhagic to better understand the temporal trend in stroke mortality in men and women.

## 5. Conclusions

In summary, there was sex disparity in the stroke proportional mortality time series, with no parallelism and coincidence in the pairwise comparison between men and women. The mortality rates’ time series for men and women occurred parallel and coincidentally. These findings underscore the importance of equity between the sexes in healthcare and highlight the need to take action to prevent stroke-related deaths.

## Figures and Tables

**Table 1:** Number of deaths, mortality rate, and proportional mortality from stroke, stratified by sex, in the metropolitan area of Greater Vitoria, Brazil, from 2000 to 2021.

	Number of Deaths		Mortality Rate (per 100,000 Inhabitants)		Proportional Mortality	
	Men	Women	Men	Women	Men	Women
2000	354	318	83.1	67.3	8.0	11.4
2001	325	295	74.1	60.6	7.3	10.1
2002	339	343	75.1	68.4	7.6	11.9
2003	319	321	68.7	62.3	6.7	10.4
2004	296	282	62.1	53.3	6.1	9.1
2005	227	279	46.4	51.3	4.7	8.9
2006	268	272	53.2	48.7	5.4	8.0
2007	235	280	45.3	48.8	4.6	8.3
2008	227	253	42.6	42.9	4.4	7.1
2009	230	233	42.0	38.5	4.4	6.2
2010	268	294	47.8	47.5	5.0	7.8
2011	230	254	40.1	40.1	4.4	6.7
2012	235	274	40.1	42.3	4.4	7.1
2013	225	229	37.6	34.7	4.2	5.9
2014	221	228	36.2	33.8	4.2	5.6
2015	222	231	35.7	33.6	4.2	5.5
2016	213	226	33.6	32.2	3.9	5.2
2017	222	205	34.3	28.7	3.8	4.4
2018	238	256	36.1	35.1	4.1	5.6
2019	306	364	45.5	49.0	5.2	7.3
2020	325	370	47.5	49.0	4.3	6.1
2021	327	347	46.9	45.1	4.0	4.9

**Table 2:** Annual percentage change, average annual percentage change, and pairwise comparison in the mortality rate (per 100,000 inhabitants) due to stroke stratified by sex from 2000 to 2021.

	Trend					Pairwise Comparison			
	Segment	APC	*p*	AAPC	*p*	AAPC Difference	*p*	Coincidence *p*	Parallelism *p*
						1.3	0.274	0.093	0.114
Men									
	2000–2021			−2.4	<0.001				
	2000–2007	−8.4	<0.001						
	2007–2017	−2.9	0.008						
	2017–2021	10.3	0.011						
Women									
	2000–2021			−1.1	0.156				
	2000–2017	−4.5	<0.001						
	2017–2021	14.4	0.003						

APC: annual percentage change; AAPC: average annual percentage change; AAPC difference: difference in annual percentage change between the sexes.

**Table 3:** Annual percentage change, average annual percentage change, and pairwise comparison in proportional mortality from stroke stratified by sex from 2000 to 2021.

	Trend					Pairwise Comparison			
	Segment	APC	*p*	AAPC	*p*	AAPC Difference	*p*	Coincidence *p*	Parallelism *p*
						0.2	0.869	<0.001	0.003
Men									
	2000–2021			−3.3	0.002				
	2000–2016	−8.3	<0.001						
	2016–2021	−0.7	0.254						
Women									
	2000–2021			−3.1	<0.001				
	2000–2007	−4.9	<0.001						
	2007–2021	2.8	0.466						

APC: annual percentage change; AAPC: average annual percentage change; AAPC difference: difference in annual percentage change between the sexes.

## Data Availability

Data were extracted from: https://datasus.saude.gov.br/informacoes-de-saude-tabnet/ (accessed on 10 November 2023).

## References

[1] Feigin V.L., Brainin M., Norrving B., Martins S., Sacco R.L., Hacke W., Fisher M., Pandian J., Lindsay P. (2022). World Stroke Organization (WSO): Global Stroke Fact Sheet 2022. Int. J. Stroke.

[2] Feigin V.L., Stark B.A., Johnson C.O., Roth G.A., Bisignano C., Abady G.G., Abbasifard M., Abbasi-Kangevari M., Abd-Allah F., Abedi V. (2021). Global, Regional, and National Burden of Stroke and Its Risk Factors, 1990–2019: A Systematic Analysis for the Global Burden of Disease Study 2019. Lancet Neurol..

[3] Naghavi M., Ong K.L., Aali A., Ababneh H.S., Abate Y.H., Abbafati C., Abbasgholizadeh R., Abbasian M., Abbasi-Kangevari M., Abbastabar H. (2024). Global Burden of 288 Causes of Death and Life Expectancy Decomposition in 204 Countries and Territories and 811 Subnational Locations, 1990–2021: A Systematic Analysis for the Global Burden of Disease Study 2021. Lancet.

[4] Soto Á., Guillén-Grima F., Morales G., Muñoz S., Aguinaga-Ontoso I., Vanegas J. (2022). Trends in Mortality from Stroke in Latin America and the Caribbean, 1979–2015. Glob. Heart.

[5] Ouriques Martins S.C., Sacks C., Hacke W., Brainin M., de Assis Figueiredo F., Marques Pontes-Neto O., Lavados Germain P.M., Marinho M.F., Hoppe Wiegering A., Vaca McGhie D. (2019). Priorities to Reduce the Burden of Stroke in Latin American Countries. Lancet Neurol..

[6] Djaló A.C.N., de Souza O.F., Maud H., Cavalcanti M.P.E., Pereira G.D.A.V., Campos M.F., Figueiredo J.L. (2024). Mortality from Cerebral Stroke in the State of Pernambuco, Brazil: An Ecological Study. J. Hum. Growth Dev..

[7] Roni G., Araújo A.C.B., Maud H., Noll M., de Souza H.M., Campos M.F., de Souza O.F. (2024). Mortality from Stroke in Pará, Brazilian Amazon: A Joinpoint Analysis. J. Hum. Growth Dev..

[8] Wang W., Wang D., Liu H., Sun H., Jiang B., Ru X., Sun D., Chen Z., Wang Y. (2017). Trend of Declining Stroke Mortality in China: Reasons and Analysis. Stroke Vasc. Neurol..

[9] Ananth C.V., Brandt J.S., Keyes K.M., Graham H.L., Kostis J.B., Kostis W.J. (2023). Epidemiology and Trends in Stroke Mortality in the USA, 1975–2019. Int. J. Epidemiol..

[10] Dinç G., Sözmen K., Gerçeklioğlu G., Arık H., Critchley J., Ünal B. (2013). Decreasing Trends in Cardiovascular Mortality in Turkey between 1988 and 2008. BMC Public Health.

[11] Cayuela A., Cayuela L., Ortega Belmonte M.J., Rodríguez-Domínguez S., Escudero-Martínez I., González A. (2022). Has Stroke Mortality Stopped Declining in Spain?. Neurol. Engl. Ed..

[12] da Silva Paiva L., Oliveira F.R., de Alcantara Sousa L.V., Dos Santos Figueiredo F.W., de Sá T.H., Adami F. (2019). Decline in Stroke Mortality Between 1997 and 2012 by Sex: Ecological Study in Brazilians Aged 15 to 49 Years. Sci. Rep..

[13] Shajahan S., Sun L., Harris K., Wang X., Sandset E.C., Yu A.Y., Woodward M., Peters S.A., Carcel C. (2023). Sex Differences in the Symptom Presentation of Stroke: A Systematic Review and Meta-Analysis. Int. J. Stroke Off. J. Int. Stroke Soc..

[14] Ali M., van Os H.J.A., van der Weerd N., Schoones J.W., Heymans M.W., Kruyt N.D., Visser M.C., Wermer M.J.H. (2022). Sex Differences in Presentation of Stroke: A Systematic Review and Meta-Analysis. Stroke.

[15] Reeves M.J., Bushnell C.D., Howard G., Gargano J.W., Duncan P.W., Lynch G., Khatiwoda A., Lisabeth L. (2008). Sex Differences in Stroke: Epidemiology, Clinical Presentation, Medical Care, and Outcomes. Lancet Neurol..

[16] de Melo Lucena D.M., Dos Santos Figueiredo F.W., de Alcantara Sousa L.V., da Silva Paiva L., do Carmo Almeida T.C., Galego S.J., Correa J.A., da Silva Maciel E., Adami F. (2018). Correlation between Municipal Human Development Index and Stroke Mortality: A Study of Brazilian Capitals. BMC Res. Notes.

[17] Wu S.H., Woo J., Zhang X.-H. (2013). Worldwide Socioeconomic Status and Stroke Mortality: An Ecological Study. Int. J. Equity Health.

[18] Reis M.F.D., Chaoubah A. (2023). The Burden of Stroke in the Southeast Region of Brazil in 2019: An Estimate Based on Secondary Data from the Brazilian United Health System. Int. J. Cardiovasc. Sci..

[19] Programa das Nações Unidas para o Desenvolvimento Atlas Brasil. http://atlasbrasil.org.br/perfil/rm/63200.

[20] Joinpoint Regression Program. https://surveillance.cancer.gov/joinpoint/.

[21] da Silva Paiva L., de Alcantara Sousa L.V., Oliveira F.R., de Carvalho L.E.W., Raimundo R.D., Correa J.A., de Abreu L.C., Adami F. (2022). Temporal Trend of the Prevalence of Modifiable Risk Factors of Stroke: An Ecological Study of Brazilians between 2006 and 2012. Int. J. Environ. Res. Public Health.

[22] de Carvalho Bastone A., de Souza Moreira B., de Souza Vasconcelos K.S., Magalhães A.S., Coelho D.M., da Silva J.I., Bezerra V.M., dos Santos Lopes A.A., de Lima Friche A.A., Caiaffa W.T. (2022). Time Trends of Physical Activity for Leisure and Transportation in the Brazilian Adult Population: Results from Vigitel, 2010–2019. Cad. Saúde Pública.

[23] NCD Risk Factor Collaboration (NCD-RisC) (2021). Worldwide Trends in Hypertension Prevalence and Progress in Treatment and Control from 1990 to 2019: A Pooled Analysis of 1201 Population-Representative Studies with 104 Million Participants. Lancet.

[24] Velek P., Splinter M.J., Ikram M.K., Ikram M.A., Leening M.J.G., van der Lei J., Olde Hartman T., Peters L.L., Tange H., Rutten F.H. (2022). Changes in the Diagnosis of Stroke and Cardiovascular Conditions in Primary Care During the First 2 COVID-19 Waves in the Netherlands. Neurology.

[25] Nogueira R.G., Qureshi M.M., Abdalkader M., Martins S.O., Yamagami H., Qiu Z., Mansour O.Y., Sathya A., Czlonkowska A., Tsivgoulis G. (2021). Global Impact of COVID-19 on Stroke Care and IV Thrombolysis. Neurology.

[26] Cougo P., Besen B., Bezerra D., Moreira R.C., Brandão C.E., Salgueiro E., Balduino A., Pontes-Neto O., Cravo V. (2022). Social Distancing, Stroke Admissions and Stroke Mortality During the COVID-19 Pandemic: A Multicenter, Longitudinal Study. J. Stroke Cerebrovasc. Dis. Off. J. Natl. Stroke Assoc..

[27] Narrett J.A., Mallawaarachchi I., Aldridge C.M., Assefa E.D., Patel A., Loomba J.J., Ratcliffe S., Sadan O., Monteith T., Worrall B.B. (2023). Increased Stroke Severity and Mortality in Patients with SARS-CoV-2 Infection: An Analysis from the N3C Database. J. Stroke Cerebrovasc. Dis. Off. J. Natl. Stroke Assoc..

[28] Pujol-Lereis V.A., Flores A., Barboza M.A., Abanto-Argomedo C., Amaya P., Bayona H., Bonardo P., Diaz-Escobar L., Gomez-Schneider M., Góngora-Rivera F. (2021). COVID-19 Lockdown Effects on Acute Stroke Care in Latin America. J. Stroke Cerebrovasc. Dis..

[29] da Silva L.G., Bezerra I.M.P., Santos G.L., Abreu L.C. (2024). de Comparative Analysis of Epidemiological Outcome of Incidence, Mortality and Lethality by COVID-19 between the States of Espírito Santo and Minas Gerais, Brazil. Epidemiologia.

[30] Santos G.L., Morais T.C., da Rocha J.B.F., da Silva L.G., do Nascimento Moratti E., Sampaio S.A., de Abreu L.C. (2023). COVID-19 in Rio de Janeiro, Brazil: A Perspective on Epidemiological Events. J. Hum. Growth Dev..

[31] da Silva A.P., Ribeiro M.A., Emídio M.P., Daboin B.E.G., Morais T.C., Mesaroch A., de Souza I.S., de Oliveira Abreu C.I.P., Bezerra I.M.P., de Abreu L.C. (2022). COVID-19 in the Municipalities of Botucatu and Serrana, São Paulo, Brazil, the Effects of Lethality and Mortality. J. Hum. Growth Dev..

[32] Rexrode K.M., Madsen T.E., Yu A.Y.X., Carcel C., Lichtman J.H., Miller E.C. (2022). The Impact of Sex and Gender on Stroke. Circ. Res..

[33] Demel S.L., Kittner S., Ley S.H., McDermott M., Rexrode K.M. (2018). Stroke Risk Factors Unique to Women. Stroke.

[34] Bushnell C.D., Kapral M.K. (2023). Stroke in Women and Unique Risk Factors. Stroke.

[35] Appelros P., Stegmayr B., Terént A. (2009). Sex Differences in Stroke Epidemiology. Stroke.

[36] Phan H.T., Gall S.L., Blizzard C.L., Lannin N.A., Thrift A.G., Anderson C.S., Kim J., Grimley R., Castley H.C., Hand P. (2019). Sex Differences in Care and Long-Term Mortality After Stroke: Australian Stroke Clinical Registry. J. Women’s Health.

[37] Instituto Brasileiro de Geografia e Estatística Tabela 7362: Esperança de Vida Ao Nascer e Taxa de Mortalidade Infantil, Por Sexo. https://sidra.ibge.gov.br/tabela/7362#resultado.

[38] Instituto Brasileiro de Geografia e Estatística Tabela 7358: População, Por Sexo e Idade. https://sidra.ibge.gov.br/tabela/7358#resultado.

[39] de Macedo Cardoso M.L., Costa P.P., Costa D.M., Xavier C., Souza R.M.P. (2017). The National Permanent Health Education Policy in Public Health Schools: Reflections from Practice. Ciênc. Saúde Coletiva.

[40] Stavseth M.R., Clausen T., Røislien J. (2019). How Handling Missing Data May Impact Conclusions: A Comparison of Six Different Imputation Methods for Categorical Questionnaire Data. SAGE Open Med..

[41] Carvalho Malta D., Sardinha L., Moura L., Lansky S., Leal M., Szwarcwald C., França E., Almeida M., Duarte E., Pedrosa A. (2010). Update of Avoidable Causes of Deaths Due to Interventions at the Brazilian Health System. Epidemiol. E Serviços Saúde.

